# Psoralen Promotes Proliferation, Migration, and Invasion of Human Extravillous Trophoblast Derived HTR-8/Svneo Cells *in vitro* by NF-κB Pathway

**DOI:** 10.3389/fphar.2022.804400

**Published:** 2022-04-08

**Authors:** Dan Qi, Jingyuan Lu, Ziyi Fu, Shanshan Lv, Lili Hou

**Affiliations:** ^1^ Department of Traditional Chinese Medicine, Nanjing Maternity and Child Health Care Hospital, Women’s Hospital of Nanjing Medical University, Nanjing, China; ^2^ Department of Radiological Intervention, Nanjing Maternity and Child Health Care Hospital, Women’s Hospital of Nanjing Medical University, Nanjing, China

**Keywords:** psoralen, proliferation, migration, invasion, human extravillous trophoblast, NF-κB pathway

## Abstract

Recurrent spontaneous abortion (RSA) is a kind of pathological pregnancy, and abnormal function of trophoblast cells may be related to a variety of pregnancy complications including RSA. Psoralen is an effective ingredient extracted from *Cullen corylifolium* (L.) Medik. with multiple bioactivities mainly including anti-osteoporotic, anti-tumor, anti-inflammatory, and estrogen-like effects. However, the exact role of psoralen on trophoblast invasiveness has not been investigated thus far. In the present study, the effects of psoralen on the proliferation, migration, and invasion abilities of HTR-8/SVneo cells were evaluated by the CCK-8 and Transwell assays. The expression patterns of nuclear factor κB (NF-κB)/p65 and metalloproteinases (MMP)-2 and MMP-9 were characterized by further experiments including real-time quantitative polymerase chain reaction and Western blot. Indirect immunofluorescence was applied to track the NF-κB p65 translocation. Herein, we found that cell viability and invasive ability were promoted by psoralen in a concentration-dependent manner. Psoralen concentration-dependently enhanced both MMP-2 and MMP-9 expression and their activity of HTR-8/SVneo cells. Additionally, we observed accelerated nuclear accumulation and enhanced nuclear translocation of p65 in the presence of psoralen. Furthermore, invasiveness enhancement of psoralen on HTR-8/SVneo cells was partly eliminated by a NF-κB pathway inhibitor. Thus, our findings suggest that psoralen may serve as a potential repurpose drug candidate that can be used to induce migration and invasion of trophoblast cells through strengthening the NF-κB pathway.

## Introduction

Recurrent spontaneous abortion (RSA) is a kind of pathological pregnancy, referring to three or more consecutive spontaneous miscarriages with the same sexual partner before 20 weeks of gestation (La et al., 2021). Although the incidence of RSA is less than 5% among women of childbearing age, it seriously endangers women’s reproductive health and imposes considerable physical and mental burdens for patients (Zhang et al., 2016; Koert et al., 2019). The possible causes of RSA include genital tract infection, chromosome abnormality, abnormal anatomy of genital tract, endocrine disturbance, immune disorders, etc. (Lanasa and Hogge, 2000; Nigro et al., 2011; Yang et al., 2018). However, the etiology of 50% of RSA patients is still unclear. Due to the complexity and heterogeneity of RSA etiology and the lack of effective treatment strategies, it is difficult to prevent and cure RSA in gynecology and obstetrics. Therefore, it is pivotal to understand the pathogenesis of RSA and more effective strategies are necessary to better manage this frustrating disease.

Establishment of pregnancy requires the implantation of fertilized ovum into the receptive endometrium, so as to promote the formation of normal placenta (Niringiyumukiza et al., 2018). Abnormal implantation and placental formation are the main causes of infertility, and can lead to early abortion (Cordo-Russo et al., 2009). Studies have shown that the development of trophoblast is very important for embryo implantation and pregnancy maintenance (Staud and Karahoda, 2018; Huppertz, 2019; Knöfler et al., 2019). Moreover, angiogenic factors such as endocrine gland-derived VEGF that could affect placental growth and trophoblast invasion, thus providing a link between abnormal placentation and insufficient trophoblast infiltration (Brouillet et al., 2013). However, abnormal function of trophoblast cells may be related to a variety of pregnancy complications including RSA (Sun and Zhang, 2017; Huppertz, 2019). Especially, the insufficient infiltration of trophoblasts leads to the transformation disorder of spiral arterioles, which further aggravates the remodeling disorder of placental vessels, leading to placental ischemia and hypoxia (Staud and Karahoda, 2018; Knöfler et al., 2019). Therefore, it is speculated that improving infiltration capacity of trophoblast cells may be a feasible way to increase the successful rate of pregnancy for RSA patients.

There are abundant ancient and modern literature records on the understanding and treatment of recurrent abortion in traditional Chinese medicine (Hullender Rubin, et al., 2013; Li et al., 2020). Traditional Chinese medicine, bioactive natural products containing special ingredients with varieties of pharmacological activities, shows a great therapeutic potential for treating various miscarriages including RSA (Zhu et al., 2014; Li et al., 2020; Cao et al., 2021). With the development of separation, purification, and preparation of effective components of Chinese herbal medicine, great progress has been made in the study of Chinese medicine monomers for RSA (Wang et al., 2021). Psoralen, the main chemical component identified in the seeds of *Cullen corylifolium* (L.) Medik., has many bioactivities. For example, Wang et al. (2019) reported a novel therapeutic potential of psoralen for the prevention and treatment of hepatocellular carcinoma. Another study suggested a protective effect of psoralen on chondrocytes, thereby exhibiting anti-inflammatory effects on synoviocytes, and attenuating monosodium iodoacetate-induced osteoarthritis (Wang C. et al., 2019). Besides, research by Liu et al. (2021) highlighted the potential ability of psoralen to act as a novel natural agent to prevent and treat periodontitis. The above evidence suggests that the functions of psoralen are related to cell proliferation and invasion. Therefore, it is speculated that psoralen may regulate the infiltration capacity of trophoblast cells.

Accumulating evidence has proved that matrix metalloproteinases (MMPs) are closely related to the invasion of trophoblast cells (Cabral-Pacheco et al., 2020). Among many MMPs, MMP-2 and MMP-9 are the main proteolytic enzymes facilitating trophoblastic invasion to the endometrium, thereby regulating the implantation process and development of embryos (Staun-Ram et al., 2004; Suo et al., 2020). Studies have shown that nuclear factor κB (NF-κB) activation can enhance the expression of MMP-2 and MMP-9 (Wang et al., 2014; Huang et al., 2017) and improve the invasive capability of trophoblast cells (Cheng et al., 2019; Oh et al., 2020; Wu et al., 2020), linking it to embryo implantation and placental formation. Of note, a study by Hu et al. (2018) confirmed the decreased expression of NF-κB in RSA patients compared with women with healthy pregnancies. Attempts of modifying NF-κB activation may improve the inadequate invasion and migration of trophoblast cells, for instance, knockdown of Notch-1 inhibited migration and invasion, down-regulated MMPs, and suppressed NF-κB signaling pathway in trophoblast cells (Yu et al., 2014). Autophagy inhibition facilitated the invasiveness of trophoblast cells by enhancing NF-κB activity (Oh et al., 2020). An experimental result showed that NF-κB pathway had participated in the regulation effect of MIR503HG on invasion and migration of trophoblast cells (Cheng et al., 2019). Obviously, NF-κB-mediated MMP-2/9 activation has played a significant role in trophoblast invasion during the establishment of pregnancy. To our knowledge, there are rare studies and reports about the effect of traditional Chinese monomer psoralen on RSA. In view of this, we investigated the effects of psoralen on the proliferation, migration, and invasion of trophoblast cells. HTR-8/SVneo cells derived from human extravillous trophoblast were selected as the research objects. This study adopted the cell counting kit-8 (CCK-8) proliferation assay, cell migration and invasion assay (Transwell test), real time fluorescence quantitative PCR (qPCR), and Western blot to investigate the exact influence of psoralen on trophoblast cells as well as the expressions of MMP-2 and MMP-9. Finally, activity of NF-κB pathway was mentioned to unveil a possible molecular mechanism involved in the regulation of psoralen on trophoblast cell invasion and migration.

## Materials and Methods

### Preparation of Psoralen

**FIGURE 1:**
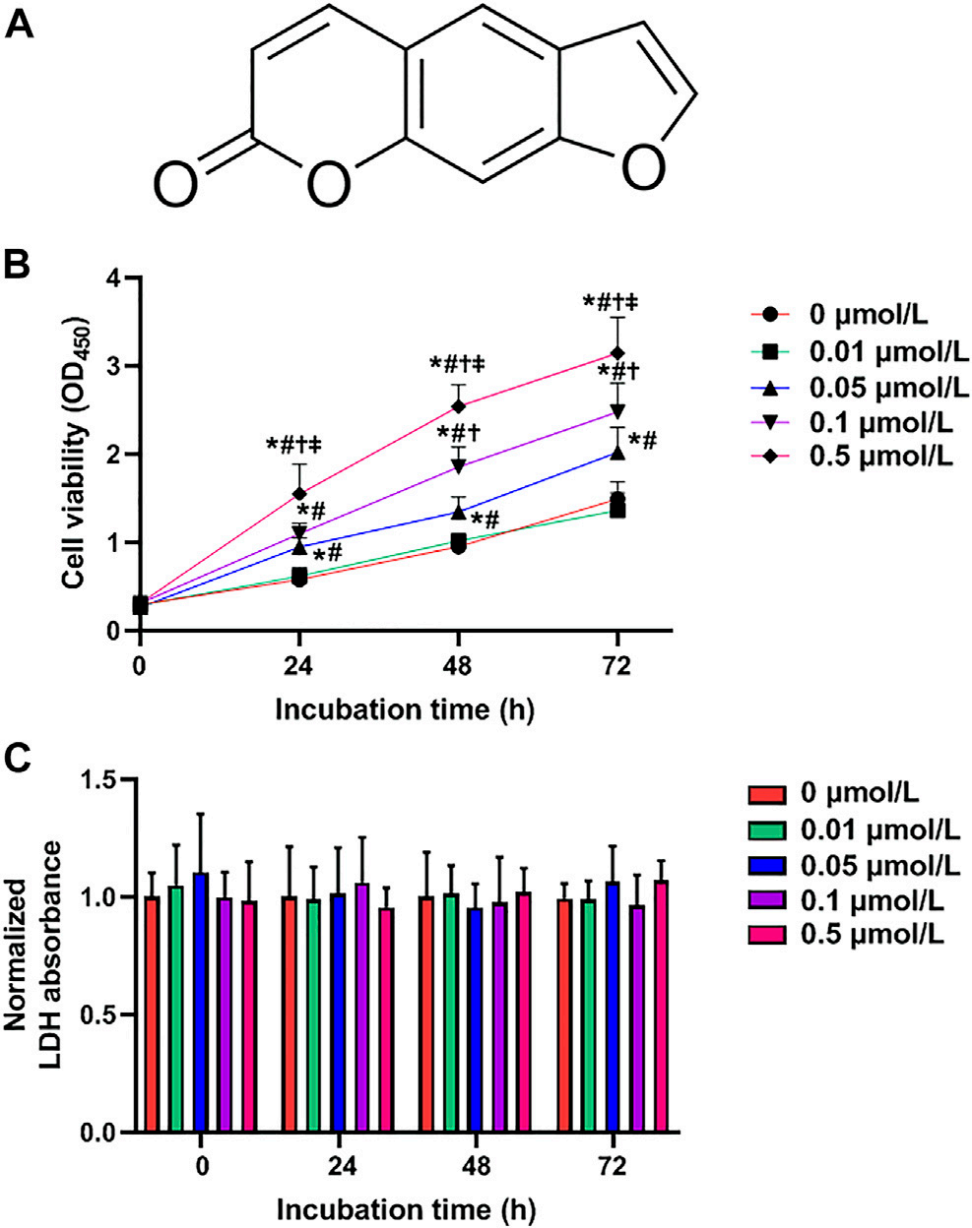
Psoralen enhances HTR-8/SVneo cell viability. **(A)** Chemical structure of psoralen. Cell viability **(B)** and LDH release **(C)** of HTR-8/SVneo cells after exposure to different concentrations (0, 0.01, 0.05, 0.1, and 0.5 μmol/L) of psoralen for 0, 24, 48, and 72 h. Values are mean ± SD. Experiments were performed in triplicate and repeated three times. ^*^ *p* < 0.05 vs. 0 μmol/L; ^#^ *p* < 0.05 vs. 0.01 μmol/L; ^†^ *p* < 0.05 vs. 0.05 μmol/L; ^‡^ *p* < 0.05 vs. 0.1 μmol/L.

Psoralen with purity over 99% by HPLC analysis (cat. P8399) was obtained from Sigma (St. Louis, MO), and its chemical structure is shown in Figure 1A. Psoralen was dissolved in dimethyl sulfoxide (DMSO) and then diluted with culture medium to yield final concentration of 0.01, 0.05, 0.1, and 0.5 μmol/L. The final concentration of DMSO in the solutions applied to cells was 0.2% (v/v). HTR8/SVneo cells in the control group were exposed only to RPMI 1640 medium supplemented with 0.2% DMSO. These concentrations of psoralen for HTR-8/SVneo cells were determined in the pre-experiment. The entire drug dispensing process was completed in a sterile environment.

### Cell Culture

The frozen human trophoblast HTR-8/SVneo cells were purchased from Shanghai Cell Bank, Chinese Academy of Sciences. After resuscitation, cells were placed in RPMI 1640 medium (Gibco-BRL, Rockville, MD) containing 10% fetal bovine serum (Hyclone, Logan, UT), 100 U/ml penicillin, and 100 μg/ml streptomycin (Sigma, St. Louise, MO) in an incubator at 37°C, 5% CO_2_, and saturated humidity. The well-grown cells at the logarithmic growth phase were collected for the subsequent experiments.

### Detection of Cell Proliferation by CCK-8

HTR-8/SVneo cells at the logarithmic growth phase were harvested and adjusted to a concentration of 4×10^3^/L. The cells were inoculated to a 96-well culture plate with an inoculum size of 100 µl per well. The adhering cells were treated with medium containing different concentrations of psoralen (0.01, 0.05, 0.1, and 0.5 μmol/L). HTR-8/SVneo cells without exposure to psoralen were chosen as a control group. For each concentration, three paralleled holes were set up. After incubation for 24, 48, and 72 h, 10-μl CCK-8 (Donjino, Japan) was added to each hole, and incubated in an incubator for 2 h. The absorbance at 450 nm (A_450_) was measured using a BioTek Synergy H4 Hybrid reader.

### Detection of Toxicity by a Lactate Dehydrogenase Assay Kit

Cell cytotoxicity was determined by quantifying LDH released from cultured cells via an LDH Assay (cytotoxicity) (#ab65393, Abcam, Cambridge, United Kingdom) according to manufacturer’s protocol. Briefly, cell culture medium was transferred into a new plate, mixed with LDH reaction mix and incubated for 30 min at room temperature. The reaction was stopped by adding a stop solution, and the absorbance at 450 nm was measured using a BioTek Synergy H4 Hybrid reader.

### Detection of Cell Migration and Invasion by Transwell Assay

The Transwell chambers with 8-μm pore size (Corning Costar, Lowell, MA) were used to detect the migration and invasion abilities of HTR-8/SVneo cells. As for Transwell migration assay, HTR-8/SVneo cell suspension (approximately 60–80×10^3^ cells) in 200 μl was added to the upper chamber. After the cells attached, the intact medium of the 10% fetal bovine serum was replaced with a serum-free medium containing psoralen (0.01, 0.05, 0.1, and 0.5 μmol/L). The lower chamber was added with 500 μl complete medium. After 24 h culture, the chamber was removed and cells on the outer surface of the membrane were subjected to fixation with 4% paraformaldehyde and staining with crystal violet. For Transwell invasion assay, the chamber was coated with the Matrigel (1:4, BD Biosciences, United States). The remaining procedure was carried out in the same way as the migration assay. Under the inverted microscope, five regions were randomly selected and photographed to count the number of cells passing through the membrane.

### Extraction of Total RNA and QPCR

After 48 h exposure to different concentrations of psoralen (0.01, 0.05, 0.1, and 0.5 μmol/L), cells were collected for the extraction of total RNA by TRIzol (Invitrogen, Carlsbad, CA). The concentration and purity of RNA were determined using a ND-1000 spectrophotometer (NanoDrop Technologies, Rockland, DE). Reverse transcription of RNA into cDNA was carried out using PrimeScript RT reagent kit with gDNA Eraser (Takara, Bio Inc., Shiga, Japan) according to the instructions of the manufacturer. Then, qPCR was performed with SYBR^®^ Green PCR master mix (Takara, Bio Inc., Shiga, Japan) using standard protocols on an ABI PRISM 7500 instrument (Applied Biosystems, United States). The GAPDH was chosen as a reference. The amplification conditions were 5 min at 95°C followed by 40 cycles of 15 s at 95°C and 1 min at 60°C. The 2^−ΔΔCt^ method was applied to determine relative expressions of MMP-2 and MMP-9. Primers were synthesized by Shanghai GenePharma Co., Ltd. (Shanghai, China), and their sequences were presented in Supplementary Table S1.

### Extraction of Protein and Western Blot Assay

Cells were harvested, lysed in RIPA buffer (Beyotime Biotech, Jiangsu, China), and the nuclear and cytoplasmic proteins were extracted, followed by the determination of their concentrations using a BCA protein assay kit (Pierce, Rockford, IL). The protein sample was separated by 10% SDS-PAGE electrophoresis and then transferred to a PVDF membrane. After being blocked with 5% non-fat milk, the membrane was incubated with the primary monoclonal antibodies (all from Santa Cruz, CA) against MMP-2 (#sc-13594), MMP-9 (#sc-393859), NF-κB/p65 (#sc-8008), and GAPDH (#sc-47724) at 4°C overnight. Two hours after incubation with the second antibody, the membrane was rinsed with PBS and visualized with enhanced chemiluminescence on X-ray film. For expression of MMP-2 and MMP-9, results were expressed as the ratio of the optical density of the interested band divided by that for GAPDH band. Levels of NF-κB/p65 between nuclear and cytoplasmic fractions were compared to evaluate the nuclear translocation activity of NF-κB.

### MMP-2/MMP-9 Activity Assay

MMP-2 and MMP-9 activity was measured by a gelatinase (MMP-2/MMP-9) Activity Assay kit (#CBA003, Calbiochem) according to the manufacturer’s protocol. Briefly, MMP-2 and MMP-9 activity of HTR-8/SVneo cells were assayed in the conditioned media after 48 h exposure to psoralen. In the assay, 90 µl of medium was removed and added to 10 µl of substrate working solution. After incubation for 18 h at 37°C in incubator with 5% CO_2_, a measurement was then performed with excitation at 320 nm and emission at 405 nm using Thermo Scientific Varioskan Flash. The MMP-2/MMP-9 activity was then expressed as the relative fluorescence unit (RFU).

### Immunofluorescence Staining

Indirect immunofluorescence and confocal laser scanning microscopy were applied to track the NF-κB p65 translocation. After incubation with psoralen (0.01, 0.05, 0.1, and 0.5 μmol/L), cells were fixed for 30 min in 4% paraformaldehyde solution at room temperature. Subsequently, they were allowed to incubate with anti-NF-κB/p65 antibody at 4°C overnight, and then with secondary anti-rabbit Alexa Fluor-labeled antibody (Life Technologies, United States) at room temperature for 2 h. Slides were stained with DAPI (Sigma, St. Louise, MO) and observed under a Nikon Microphot-FX fluorescence microscope using a ×400 magnification. Images were then processed using the ImageJ software to delimitate the nuclear region and to measure the fluorescence intensity of NF-κB p65 within the nucleus.

### Statistical Analysis

Data were analyzed using Statistical Package for Social Sciences version 20.0 (SPSS version 20, IBM Statistics, New York). Results were expressed as mean ± standard deviation (SD) and compared using one-way ANOVA with post-Turkey multiple comparison test. A *p*-value less than 0.05 was considered to indicate a statistically significant difference.

## Results

### Effects of Psoralen on Cell Viability of HTR-8/SVneo Cells

To investigate the exact effects of psoralen on growth of trophoblast cells, the CCK-8 assay was adopted to evaluate the viability of HTR-8/SVneo cells after 24, 48, and 72 h of exposure to various concentrations (0, 0.01, 0.05, 0.1, and 0.5 μmol/L) of psoralen. It was observed that psoralen at a concentration of 0.01 μmol/L caused no statistically significant effect on the viability of HTR-8/SVneo cells. In certain concentrations (0.05–0.5 μmol/L), viability of HTR-8/SVneo cells was promoted by psoralen with the increase of the concentration and treatment duration as analyzed by one-way ANOVA (Figure 1B). The main reason for the discrepancy that 0.01 μmol/L psoralen failed to cause significant effect on the cell viability was the insufficient psoralen concentration to function. In HTR-8/SVneo cells, psoralen treatment caused no cytotoxicity as evidenced by unchanged LDH release (Figure 1C).

### Effects of Psoralen on Migration Ability of HTR-8/SVneo Cells

**FIGURE 2:**
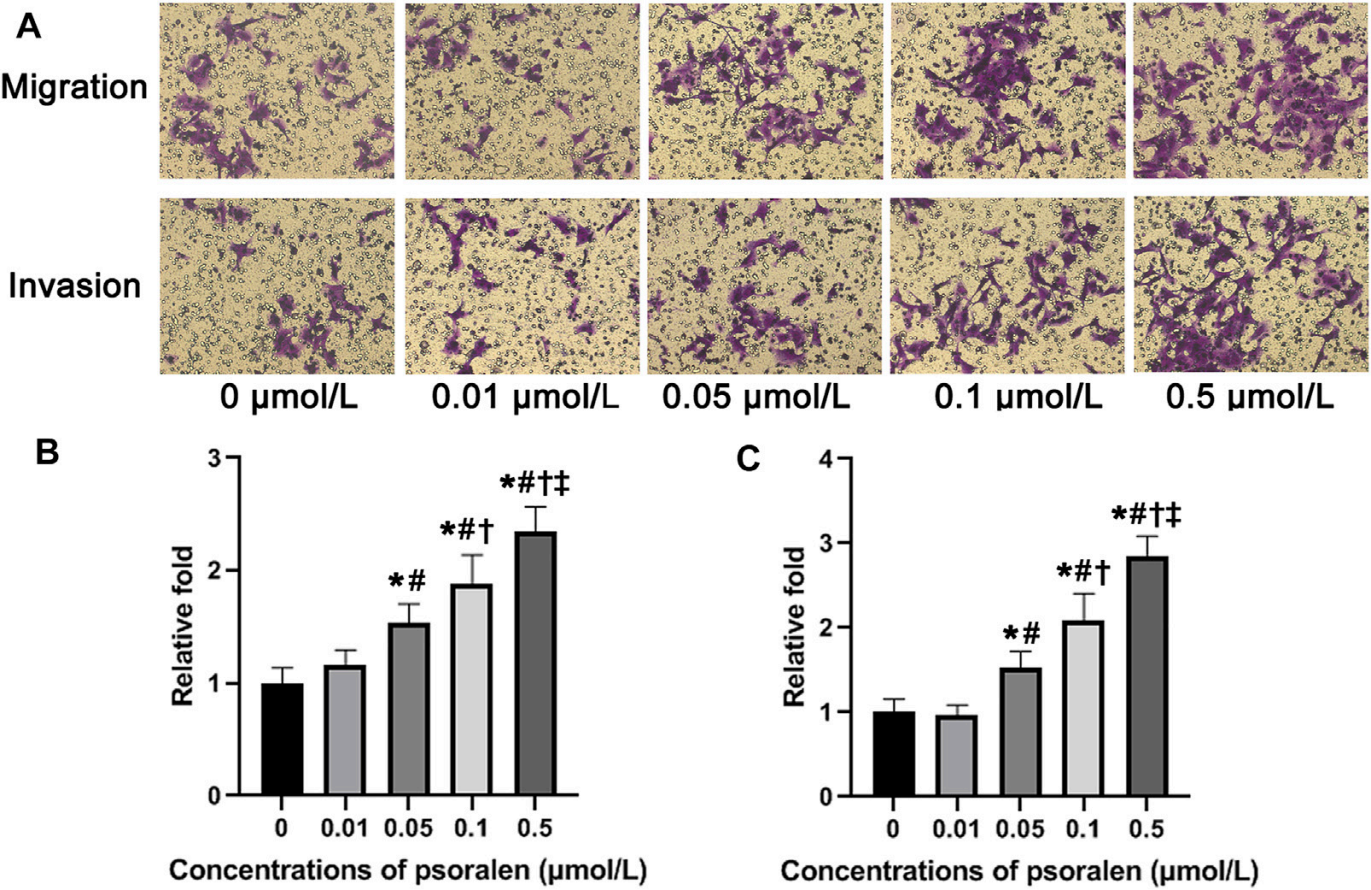
Psoralen strengthens migration and invasion ability of HTR-8/SVneo cells. **(A)** Representative images of Transwell assay of HTR-8/SVneo cells after exposure to different concentrations (0, 0.01, 0.05, 0.1, and 0.5 μmol/L) of psoralen for 24 h. **(B)** Changes in the number of migrating cells in relation to control cells. **(C)** Changes in the number of invading cells in relation to control cells. Values are mean ± SD. Experiments were performed in triplicate and repeated three times. ^*^ *p* < 0.05 vs. 0 μmol/L; ^#^ *p* < 0.05 vs. 0.01 μmol/L; ^†^ *p* < 0.05 vs. 0.05 μmol/L; ^‡^ *p* < 0.05 vs. 0.1 μmol/L.

Results from the Transwell migration assay showed a gradual rise in the number of migrating HTR-8/SVneo cells along with the increase of psoralen concentration (Figures 2A,B). Changes in the number of migrating cells exposed to psoralen at the lowest concentration (0.01 μmol/L) did not reach a statistically significant level (*p* > 0.05). The highest concentration of psoralen (0.5 μmol/L) induced the strongest migration-promoting effect on HTR-8/SVneo cells (2.3-fold increase over control, *p* < 0.05).

### Effects of Psoralen on Invasion Ability of HTR-8/SVneo Cells

According to analysis of Transwell invasion assay (Figures 2B,C), the invasion ability of HTR-8/SVneo cells showed a tendency to ascend with increasing concentration of psoralen, reaching the maximum when the concentration of psoralen was 0.5 μmol/L. Similar to the results obtained from Transwell migration assay, psoralen at a concentration of 0.01 μmol/L failed to induce statistically significant changes in the number of invading cells (*p* > 0.05).

### Effects of Psoralen on Expression and Activity of MMP-2 and MMP-9 of HTR-8/SVneo Cells

**FIGURE 3:**
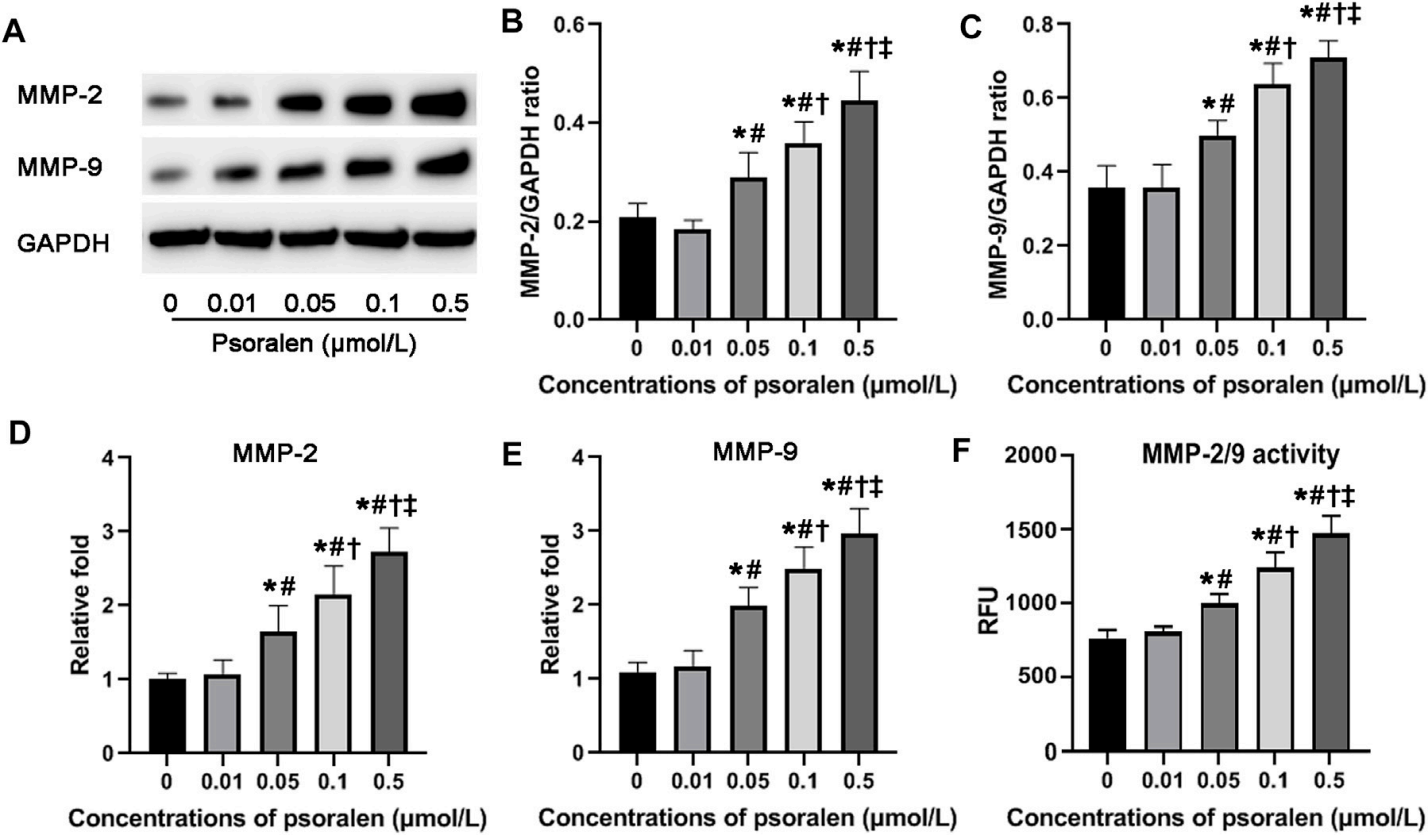
Psoralen enhances both MMP-2 and MMP-9 expression and their activity of HTR-8/SVneo cells. **(A)** Representative images of Western blot. Relative protein levels of MMP-2 **(B)** and MMP-9 **(C)** after exposure to different concentrations (0, 0.01, 0.05, 0.1, and 0.5 μmol/L) of psoralen for 48 h. Relative mRNA levels of MMP-2 **(D)** and MMP-9 **(E)** after exposure to different concentrations (0, 0.01, 0.05, 0.1, and 0.5 μmol/L) of psoralen for 48 h. **(F)** Activity of MMP-2 and MMP-9 after exposure to different concentrations (0, 0.01, 0.05, 0.1, and 0.5 μmol/L) of psoralen for 48 h. Values are mean ± SD. Experiments were performed in triplicate and repeated three times. ^*^ *p* < 0.05 vs. 0 μmol/L; ^#^ *p* < 0.05 vs. 0.01 μmol/L; ^†^ *p* < 0.05 vs. 0.05 μmol/L; ^‡^ *p* < 0.05 vs. 0.1 μmol/L.

MMPs including MMP-2 and MMP-9 are involved in cell migration and invasion under normal and pathological situations by mediating the degradation of extracellular matrix proteins. To further link the strong positive effects of psoralen on migration and invasion ability with MMP-2 and MMP-9, we then assayed their expression and activity. Results from qPCR and Western blot showed that in response to psoralen at concentrations ranging from 0.05 to 0.5 μmol/L for 48 h, mRNA and protein levels of MMP-2 and MMP-9 were significantly higher in comparison with the control (*p* < 0.05). Similarly, psoralen at certain concentrations (0.05–0.5 μmol/L) could upregulate the activity of MMP-2 and MMP-9 as indicated by activity assay kit (*p* < 0.05). The above findings suggest that psoralen enhanced both MMP-2 and MMP-9 expression and their activity (Figure 3).

### Effects of Psoralen on NF-κB Activity of HTR-8/SVneo Cells

**FIGURE 4:**
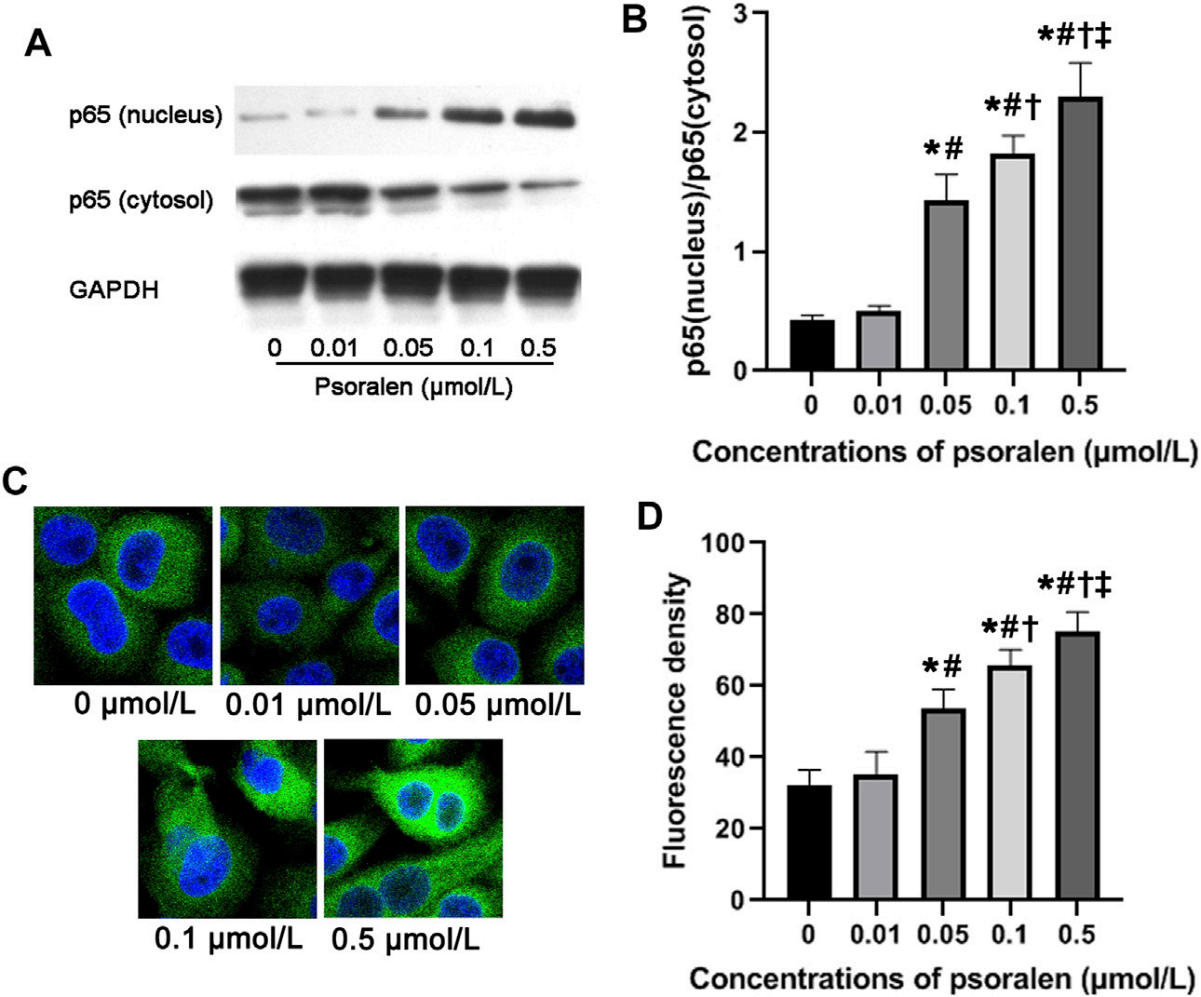
Psoralen promotes NF-κB activity of HTR-8/SVneo cells. **(A)** Representative images of Western blot. **(B)** Relative protein level of NF-κB p65 protein in HTR-8/SVneo cells after exposure to different concentrations (0, 0.01, 0.05, 0.1, and 0.5 μmol/L) of psoralen for 48 h. **(C)** Representative images of immunofluorescence staining. **(D)** Fluorescence intensity of p65 in nuclei of HTR-8/SVneo cells after exposure to different concentrations (0, 0.01, 0.05, 0.1, and 0.5 μmol/L) of psoralen for 48 h. Values are mean ± SD. Experiments were performed in triplicate and repeated three times. ^*^ *p* < 0.05 vs. 0 μmol/L; ^#^ *p* < 0.05 vs. 0.01 μmol/L; ^†^ *p* < 0.05 vs. 0.05 μmol/L; ^‡^ *p* < 0.05 vs. 0.1 μmol/L.

**FIGURE 5:**
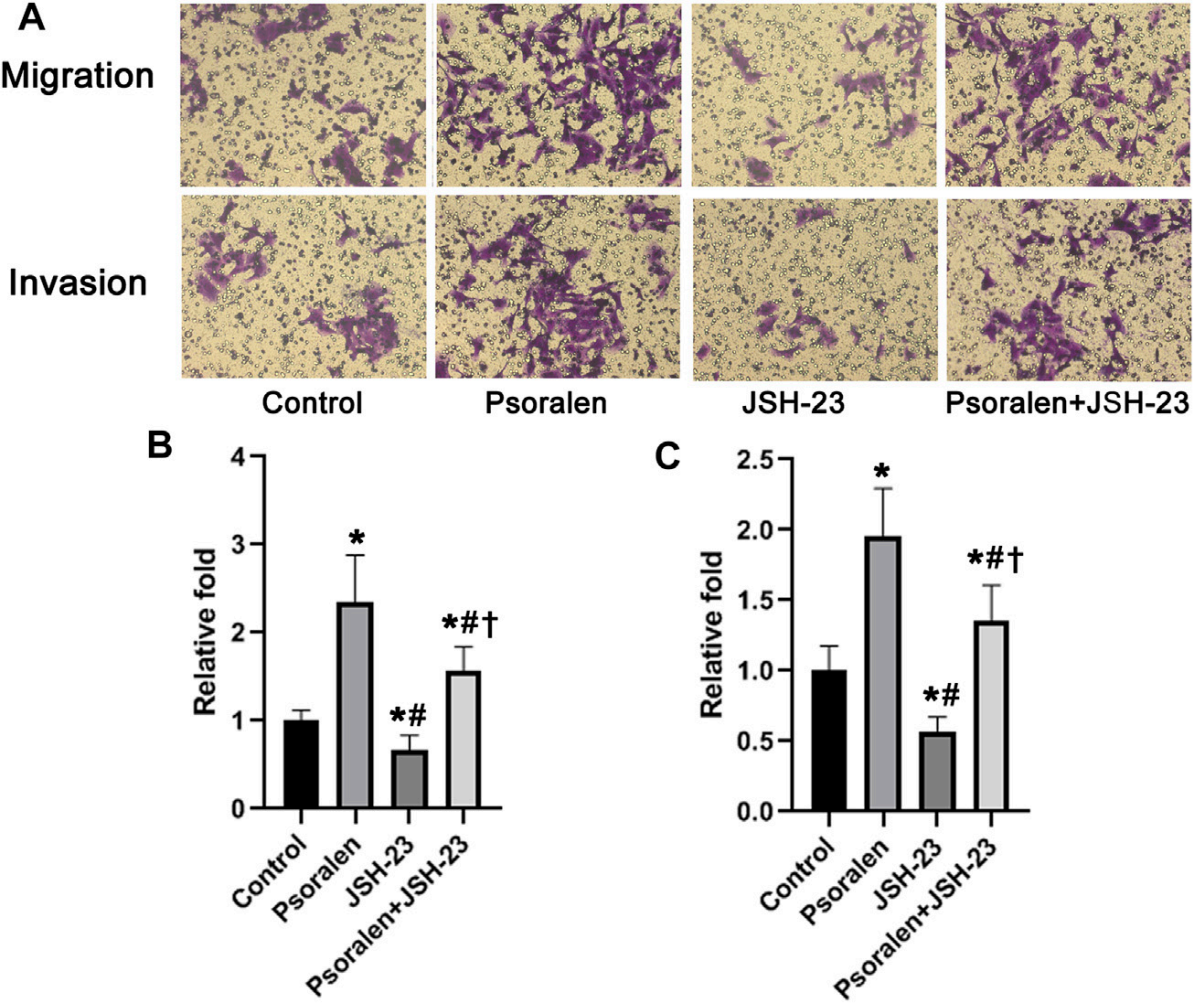
Involvement of NF-κB in psoralen-induced enhancement of cell invasiveness analyzed by Transwell assay. **(A)** Representative images of Transwell assay of HTR-8/SVneo cells after exposure to psoralen (0.5 μmol/L), JSH-23 (10 μmol/L) alone or in combination for 24 h. **(B)** Changes in the number of migrating cells in relation to control cells. **(C)** Changes in the number of invading cells in relation to control cells. Values are mean ± SD. ^*^ *p* < 0.05 vs. control; ^#^ *p* < 0.05 vs. psoralen; ^†^ *p* < 0.05 vs. JSH-23.

Mechanically, NF-κB activity has been widely reported to participate in maintaining infiltration capacity of trophoblast cells. Therefore, we further examined whether psoralen improves the trophoblast invasiveness through strengthening the NF-κB pathway. Indeed, nuclear accumulation of NF-κB p65 protein in HTR-8/SVneo cells increased in a dose-dependent manner with psoralen (Figures 4A,B). Similarly, psoralen at certain concentrations (0.05–0.5 μmol/L) could promote nuclear translocation of NF-κB p65 as indicated by immunofluorescence staining (Figures 4C,D). In addition, invasiveness enhancement of psoralen on HTR-8/Svneo trophoblast cells was partly eliminated by JSH-23 (NF-κB pathway inhibitor) as shown in Figure 5. The above findings suggested the possible involvement of NF-κB pathway in psoralen-induced invasion ability.

## Discussion

Our study reveals for the first time that psoralen has positive regulation on trophoblast functions, including proliferation, migration, and invasion. We also demonstrate that psoralen facilitates the expression and activity of MMP-2 and MMP-9, factors responsible for the cell migration and invasion, along with NF-κB activation. Furthermore, invasiveness enhancement of psoralen on HTR-8/SVneo cells is partly eliminated by an NF-κB pathway inhibitor. Collectively, these findings provide novel insight into the exact role of psoralen on invasiveness of HTR-8/SVneo trophoblast cells via NF-κB pathway and raise the intriguing possibility that psoralen may serve as a potential repurpose drug candidate used for the treatment of RSA.

RSA, an intractable infertility, has brought negative influence to women’s reproductive health and imposed a substantial burden on patients, their families, and society (Zhang et al., 2016). Although advances have been made in the understanding of this disease’s pathogenesis, difficult challenges still exist in clinical diagnosis and treatment of RSA. Emerging studies have led to significant advances in our understanding of traditional Chinese medicine especially Chinese medicine monomers in the therapy of RSA (Zhu et al., 2014). For example, this disease can be ameliorated with the use of traditional Chinese medicine preparation, including Wenjing Decoction (Hullender Rubin et al., 2013), BuShen HuoXue Decoction (Feng et al., 2020), and Tiaomian III Decoction (Gao et al., 2015). In fact, special ingredients from traditional Chinese medicine have exhibited the potential in improving the deficient trophoblast invasion. Liu et al. (2018) found that baicalein enhanced migration and invasion of extravillous trophoblasts via activation of the NF-κB pathway. According to a study by Ly et al. (2018), the phenolic compounds present in *V. angustifolium* leaf extract increased extravillous trophoblast cell migration and invasion *in vitro*. Choi et al. (2016) identified *Paeonia lactiflora* as an effective component to enhance the adhesion of trophoblast to the endometrium via induction of leukemia inhibitory factor expression. These results suggest the therapeutic potential of extracts from Chinese medicine against RSA.

Psoralen is an effective ingredient extracted from *Cullen corylifolium* (L.) Medik. with multiple bioactivities mainly including anti-osteoporotic, anti-tumor, anti-inflammatory, and estrogen-like effects (Li et al., 2017; Wang X. et al., 2019; Zhou et al., 2020; Liu et al., 2021). Recently, psoralen could reduce alveolar bone loss in experimental periodontitis possibly by affecting the intestinal immune barrier and ecological barrier and mediating immune response (Liu et al., 2021). Psoralen has been identified as a natural phytoestrogen to improve diaphyseal fracture healing in ovariectomized mice (Huang et al., 2021). According to a review by Ren et al. (2020), psoralen has been proved to be beneficial for the treatment of osteoporosis, tumors, viruses, bacteria, and inflammation. However, the exact role of psoralen on trophoblast invasiveness has not been investigated thus far. Therefore, in the present study, we intended to reveal the possible roles of psoralen in trophoblast proliferation, invasion, and migration.

Cell proliferation is the most important life characteristic and basic biological process of organism. Since the development of trophoblast is very important for embryo implantation and pregnancy maintenance (Huppertz, 2019), we first investigated the exact effects of psoralen on growth of trophoblast cells. As shown in results of CCK-8 assay, incubation with psoralen promoted the viability of HTR-8/SVneo cells in a concentration- and time-dependent manner. These earlier findings are consistent with our results showing that psoralen stimulated osteoblast proliferation by regulating NF-κB and IRE1-ASK1-JNK signaling pathway (Li et al., 2017), and chondrocyte proliferation by Wnt/β-catenin signaling pathway (Zheng et al., 2017). However, several studies regarding the role of psoralen on human cancer development have reported that exposure to psoralen caused the opposite effects on proliferation of human hepatoma SMMC7721 cells (Wang et al., 2019) and breast cancer MCF-7 and MDA-MB-231 cells (Wang et al., 2018). These contradictory results imply that psoralen regulates the cell proliferation in a cell-specific manner that it inhibits the proliferation of malignant cells while expands normal cells.

The invasive function of trophoblast cells is pivotal for the normal placentation and successful pregnancy (Carter et al., 2015; Abbas et al., 2020). In early pregnancy, trophoblast cells invade the uterus and form placental tissue with characteristics of invasiveness similar to tumor cells (Harada et al., 2007; Lunghi et al., 2007; Hammer, 2011). According to a study by Wang et al. (2018), psoralen inhibited the invasive ability of human breast cancer MCF-7/ADR cells. Indeed, our results clearly showed that psoralen promoted the invasion of trophoblast cells and enhanced the expression and activity of invasion-related molecules MMP-2 and MMP-9. The conflicting findings indicated that psoralen played different roles regarding the modulation of invasiveness under different physiological and pathological states, deepening our understanding on the properties of psoralen. To our knowledge, this is the first report demonstrating a pro-invasive effect of psoralen on trophoblast cells. An inadequacy of this study is that we assessed the effects of psoralen on the processes carried out by the HTR-8/SVneo cells such as cell proliferation, migration, and invasion. Since HTR-8/SVneo cells can develop the tube-like formation profile, further studies such as tube formation assay are needed to validate the potential of psoralen for invading trophoblasts to develop vasculogenic mimicry. NF-κB pathway, a well-studied signaling pathway (Xiao et al., 2021), is involved in regulating the invasive function of trophoblast cells (Oh et al., 2020; Wu et al., 2020). For instance, Oh et al. (2020) found that autophagy inhibition increased the invasiveness of trophoblastic cell lines by NF-κB activation as evidenced by increased NF-κB activity and p65 expression. Another study reported by Wu et al. (2020) demonstrated that nuclear receptor coactivator six promoted invasion and migration of HTR-8/SVneo cells by activating NF-κB. Moreover, Li et al. (2017) presented evidence that psoralen played its biologic role through the activation of the NF-κB pathway. In this study, we found the possible involvement of NF-κB pathway in psoralen-induced invasion ability. The above findings suggest that psoralen may promote invasive and migration capabilities of HTR-8/SVneo cells, at least partially, through NF-κB activation. NF-κB is an important transcription factor in various biological responses, and NF-κB pathway regulates many physiological cellular processes. We propose psoralen as a potential therapeutic molecule that can target specifically NF-κB pathway, and this may cause problems of specificity and side effects, related to the importance of this pathway in other physiological cellular processes. Further aspects of psoralen effects on other physiological cellular processes should be characterized. One limitation of this study was the use of an *in vitro* model in investigating effects of psoralen. To address this issue, further investigation using primary culture trophoblast cells prepared from human placenta or a mouse model are needed to validate the use of psoralen as a potential repurpose drug candidate used for the treatment of RSA.

These findings reveal that psoralen promotes the proliferation, migration, and invasion abilities of HTR-8/SVneo human trophoblasts possibly by enhancing NF-κB pathway. The results of this study deepen our understanding of the regulatory mechanism of psoralen on trophoblast function. Psoralen may serve as a potential repurpose drug candidate that can be used to induce migration and invasion of trophoblast cells. Other studies are needed to characterize the effect of this monomer on the endothelial cells. Further investigations are needed to explore how this Chinese medicine monomer might provide clinical benefits for the prevention and treatment of diseases caused by trophoblast dysfunction.

## Data Availability

All datasets generated for this study are included in the article/Supplementary Material.
