# Supplementary material for: Psoralen Promotes Proliferation, Migration, and Invasion of Human Extravillous Trophoblast Derived HTR-8/Svneo Cells in vitro by NF-κB Pathway
Source: Front Pharmacol. 2022 Apr 8;13:804400. doi: 10.3389/fphar.2022.804400 (PMC9024043; doi:10.3389/fphar.2022.804400)
Supplement: Supplementary file 1 [file Table1.docx]

Table 1: Primers used for real-time PCR

| Gene symbol | Forward primer (5’-3’) | Reverse primer (5’-3’) |
| --- | --- | --- |
| MMP-2 | TGGATGCCCTCTCTCTGGTA | AATTGTTCCCCTGCCAACCT |
| MMP-9 | AGGTTCGACGTGAAGGCG | GCCGTCCTGTACTGAAGGAG |
| GAPDH | GCAACTAGGATGGTGTGGCT | TCCCATTCCCCAGCTCTCATA |
